# The Retinal Complications of C3 Dense Deposit Disease: A Scoping Review

**DOI:** 10.3390/vision9030064

**Published:** 2025-08-01

**Authors:** Jolene McCarney, Katie Curran, Tunde Peto, Giuliana Silvestri, Laura N. Cushley

**Affiliations:** 1 Centre for Public Health, Queen’s University Belfast, Belfast BT12 6BA, UK; 2 Department of Ophthalmology, Belfast Health and Social Care Trust, Belfast BT12 6BA, UK

**Keywords:** C3 dense deposit disease, retinal disease, MPGN II, ocular complications

## Abstract

People with C3 Dense Deposit Disease (C3DDD), a rare autoimmune disease, often also have ocular complications. Due to the rarity of this disease, there is little known about ocular complications in populations across the world. This paper aimed to assess literature on retinal complications in people with C3 Dense Deposit Disease. A scoping review was conducted and three databases (Embase, Medline All, and Web of Science) were searched using agreed search terms and Boolean operators. All references were imported into Covidence for screening by two reviewers. Any conflicts were resolved by a third reviewer. Data were extracted into an Excel spreadsheet and analysis was conducted using SPSS Version 29. After full text screening, 38 studies were included in the review. These studies were from 1990–2023 and most (67%) being case reports. All studies were conducted in the United States (55%) or Europe (45%). Most studies reported drusen-like deposits in the retina (75%) and retinal pigment epithelial detachment (18%) and macular atrophy (11%). Choroidal Neovascularisation (CNV) was found in 16% of cases. People with C3 Dense Deposit Disease are at risk of ocular complications, primarily drusen-like deposits. Further population-based research and progression is needed.

## 1. Introduction

C3 dense deposit disease (C3DDD), also known as Membranoproliferative Glomerulonephritis Type II (MPGN II), is a rare autoimmune disease causing glomerular inflammation [1]. Electron dense deposits in the lamina densa of the glomerular basement membrane (GBM) are the main histopathological indicator of C3DDD [2]. GBM damage caused by these deposits results in continual loss of renal function. Approximately 50% of C3DDD patients will develop renal failure within 10 years of diagnosis [3]. Other common renal manifestations of C3DDD include haematuria, proteinuria, and hypertension [3]. C3DDD is caused by dysregulation of the alternative complement pathway, primarily of Complement Factor H (CFH). However, the exact etiology is undetermined [4]. Autoantibodies, particularly C3 nephritic factor (C3NeF), are also linked to inconsistent complement activity, with C3NeF presenting in 80% of C3DDD patients [4].

C3DDD has an incidence of approximately 2–3 individuals per million and is predominantly found in children and adolescents [1,5]. Males and females are typically equally affected by C3DDD [1]. Limited studies explore whether C3DDD varies amongst different ethnic groups or geographical locations.

Ocular complications are common amongst individuals with C3DDD [4]. A multilaminar extracellular matrix known as Bruch’s membrane (BM) separates choroidal blood vessels from the retina [6]. BM has structural and functional similarities to the GBM, and similar dense deposits are present in both structures [6]. This discovery was first documented in 1989, when an 18-year-old boy presented with renal and ocular complications including retinal pigment epithelium (RPE) detachment [7]. On examination, strikingly similar dense deposits were discovered in both his eyes and kidneys [7]. Drusen formation creates RPE elevations and regions of reflective material which is easily seen using optical coherence tomography (OCT) [8]. Often, patients retain good visual acuity and display no visual symptoms despite widespread drusen [4]. Visual loss develops due to secondary eye complications, including choroidal neovascularisation (CNV) and central serous chorioretinopathy (CSCR) [9]. Additionally, atrophic changes, especially in the macular regions, are a common finding, while retinal vascular changes are less frequent [8]. It is unclear why only some patients present with complications. Ocular manifestations of C3DDD extend beyond the retina.

Despite ongoing research into the development of drusen and other ocular manifestations, knowledge remains limited. The instigator of drusen development in C3DDD is unknown, along with the variability in drusen type and location [4]. Drusen size and location may reflect the stage of disease, however, it is uncertain if drusen is connected to disease severity [10]. Due to uncertainty surrounding severity and prevalence of ocular manifestations, the need for regular ocular complication screening is unclear [10].

At present there is no known treatment for retinal pathologies associated specifically with C3DDD, with the exception of neovascularisation by using anti-VEGF (anti-vascular endothelial growth factor) injections [11]. However, studies only focus on a singular patient, and treatments lack generalisation across large populations. The main treatment for renal complications is transplantation [3]. However, transplantation lacks long-term success and has been associated with worsening systemic effects [3].

This scoping review aims to identify existing literature on retinal pathologies associated with C3DDD.

## 2. Materials and Methods

This review was conducted according to the PRISMA-ScR guidelines [12] (Appendix A), and a protocol was followed but not registered. An information specialist (RF) was consulted to identify appropriate databases and search terms. The databases searched were MEDLINE ALL (Ovid), Embase (Ovid), and Web of Science Core Collection on 4th December 2024. Search terms for all databases were “dense deposit disease” OR “MPGN II” OR “MPGN Type 2” OR “C3DDD” AND “ocular” OR “visual” OR “retina*”. An additional Google Scholar search including ‘ocular complications in C3 Dense Deposit Disease’ and ‘Ocular Complications in MPGN Type II’ was conducted to obtain grey literature. No restrictions on study location, time/date, age, sex, or ethnicity were employed.

Eligibility criteria were (1) people with C3DDD from any population (age, gender, or ethnicity) and (2) retinal complications of C3DDD. Studies not in English, animal/in vitro studies, and conference proceedings were excluded. References were imported into Covidence systematic review software, which removed duplicates. Titles and abstracts were independently screened by two reviewers (JMcC, LNC). Discrepancies were resolved by two reviewers, and a third arbitration reviewer was consulted to resolve any further conflicts. Full texts were also screened by the same reviewers and conflicts resolved in the same way. After further conflict resolution, final texts for data extraction were obtained. One reviewer (JMcC) extracted the data into a Microsoft^®^ Excel database (Microsoft 365 for enterprise, Version 2506, Redmond, Washington, DC, USA) which was then verified by a secondary reviewer (LNC). Variables extracted included patient age, gender, and ethnicity, year and location of study, sample size, eye disease, retinal pathology, and location of pathology. The database was used to calculate frequencies and descriptive statistics of (1) patient age, gender, and ethnicity, (2) study design, sample size, and year and location of studies, and (3) ocular impacts including eye disease, retinal pathology, and location of pathology.

## 3. Results

**Figure 1:**
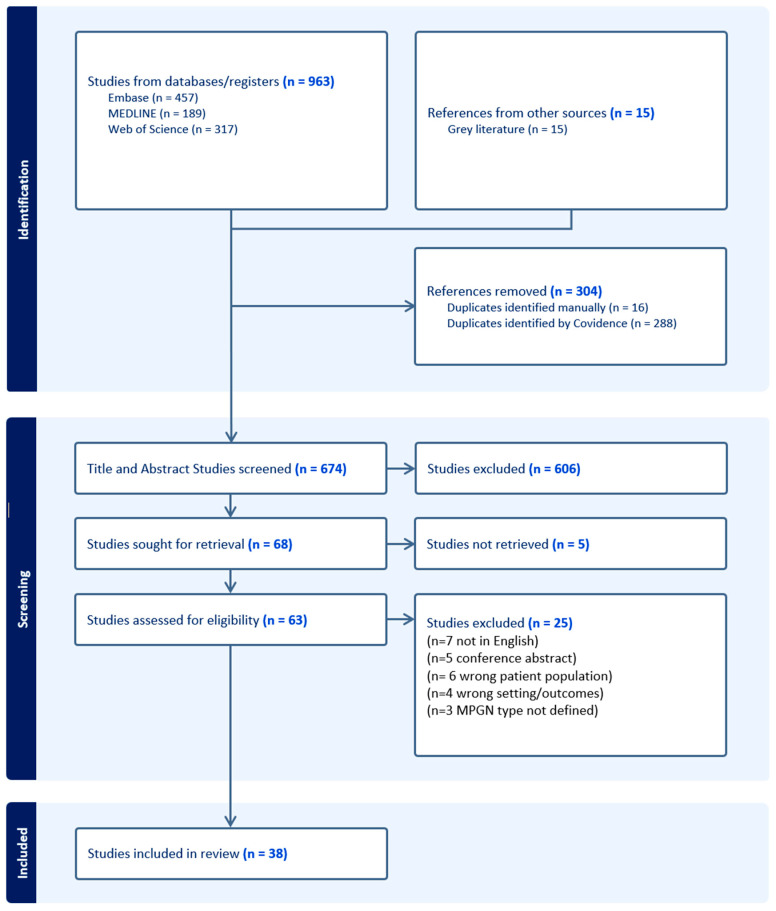
PRISMA flow chart of eligible studies for scoping review.

The search yielded 963 articles, and an additional 15 articles were identified from Google Scholar. Covidence removed 300 duplicates (31%) and all 15 papers from Google Scholar were manually removed as duplicates. Therefore, 663 titles and abstracts were screened, of which 595 (90%) were removed. Sixty-eight full texts were screened, of which thirty (38%) were removed. In total, 38 full texts were included. In accordance with PRISMA-ScR guidelines, a PRISMA flowchart was created (Figure 1).

### 3.1. Characteristics of Included Reports

This scoping review included 38 studies [3,4,8,9,10,11,13,14,15,16,17,18,19,20,21,22,23,24,25,26,27,28,29,30,31,32,33,34,35,36,37,38,39,40,41,42,43,44], published from 1990–2023. Most papers (68%) were published before 2015 (n = 26). Only 12 papers were published after 2015. Thirty-five (92%) studies were case reports, and three were retrospective case series [9,28,31]. The sample size in each report ranged from 1 patient to 26, with most (67%; n = 24) focusing on one patient. Twenty-one studies were conducted in the United States (US), and seventeen were conducted throughout Europe (Table 1). Only four papers (11%) assessed the prevalence of C3DDD [9,24,31,39].

### 3.2. Patient Population

This review identified 139 individuals with C3DDD and retinal complications. Just over half of the individuals were female (55%; n = 76), while 63 (45%) were male. The mean and median age was 39, and ages ranged from 13 to 78. The age of males and females was almost identical. Only six ethnicities were indicated; while 33 papers (84%) did not specify ethnicity, the remaining 6 studies included patients of Caucasian ethnicity (16%).

Visual Acuity (VA) at patient presentation was reported in 33 studies (86.8%), while it was not stated in a total of 5 studies, an overview can be seen in Table 2. One paper reported a mean of 0.7 and 0.8 right and left eye. One paper stated that 1 person had severe visual impairment, 1 was impaired, and 5 had normal vision. Below is a full table of VAs reported within case reports.

Many (40%) had a normal visual acuity and 6% had a slightly reduced visual acuity (6/7.5, 0.1 LogMAR). The majority (78%) had visual acuities better than 0.3 LogMAR (6/12). There was a small number of worse acuities, with 4 reportedly having a visual acuity of 1.0 logMAR (6/60) and 3 reporting counting fingers (CF).

### 3.3. Eye Disease

Several individuals exhibited diagnosed eye disorders such as central serous chorioretinopathy (CSCR), which presented in five individuals (4%). CSCR was predominantly present in males (80%; n = 4). Retinopathy was present in eight individuals (6%); seven had hypertensive retinopathy and one had Purtscher-like retinopathy. Conditions including maculopathy, scotoma, and glaucoma were rare and only reported in 1–3 patients, (Table 3). Maculopathy was described as ‘active maculopathy’ with serous detachment of the retina in one case and maculopathy with intrafoveolar subretinal neovascular membrane in the other.

### 3.4. Reported Symptoms

Some of the symptoms reported were nyctalopia (night blindness) in seven individuals (5%), a scotoma in three (2%), and photophobia in two (1%) of individuals.

### 3.5. Retinal Pathologies: Prevalence and Location

Fourteen retinal pathologies were identified, ranging from common (75%) to rare (0.7%) (Table 4).

Drusen-like deposits were the most common finding, affecting 103 individuals (75%) and most drusen were basal laminar (95%). Deposits were predominantly in the inferior retinal (32%) and macular (31%) regions. Peripheral drusen was present in 24 individuals (23%). Twelve individuals (9%) exhibited drusen scattered across the retina. Drusen was additionally detected in the posterior pole (13%), beneath the fovea (2%), and within BM (6%) and the RPE (5%).

Choroidal neovascularisation (CNV) was found in 22 individuals (16%).

RPE detachment affected 24 (18%) individuals. Some individuals (n = 13; 9%) exhibited RPE elevations and five exhibited RPE mottling (4%). Retinal pigment migration was present in four individuals (3%). Ten individuals (7%) experienced subretinal fluid underlying the RPE. Intraretinal fluid was detected in two individuals (1%). Seventeen patients displayed macular atrophy (12%). BM irregularities, including thickening and breaks, presented in 15 individuals (11%).

Retinal haemorrhages were found in 15 individuals (11%); most were subretinal (40%). Four of the subretinal haemorrhages were substantial and the other two were small, although no specific measurements were given. Three individuals had extrafoveal haemorrhages. Additional haemorrhages included one intraretinal, one macular, and one preretinal. Two individuals (1%) had retinal vein occlusions. One individual (0.7%) displayed attenuated retinal arteries.

### 3.6. Treatment

Of the 38 studies, 9 (23.7%) described treatments of patients within the reports. Three had been treated with anti-VEGF injections, three had been treated with laser, and one patient was treated with laser and injections. One patient was treated with vitrectomy and cataract surgery and one with trabeculectomy (not related to C3DDD). A total of 26 studies did not describe any ocular treatments; however, three described other treatments such as weight loss or kidney transplant. Those treated with anti-VEGF injections had CNV present (two cases) and macular oedema (one case). The other patient treated with injections, laser, and photodynamic therapy has a recurrent submacular CNV and subretinal haemorrhage. The cases with laser treatment had chorodal neovascularization (2 cases) and degenerative retinoschisis and micro-cystoid degeneration [1].

## 4. Discussion

C3DDD is a rare, progressive disease resulting in renal failure [1]. Ocular pathologies are commonly identified in C3DDD patients. Clinical manifestations range from asymptomatic to detrimental visual impairment [4]. This review focuses on retinal complications of C3DDD. We highlight specific ocular pathologies associated with C3DDD, primarily drusen-like deposits and CNV, while the others affect different retinal structures.

This review indicates that ocular manifestations of C3DDD affect males and females nearly equally [1], although one study did report a female preponderance of 3:1 [34]. The patients are predominantly in the age group where long-term outcomes matter the most. Unfortunately, due to lack of reported information, we are unable to analyse ethnic differences. The majority of the reported cases were white Caucasian.

As there is no screening for this disease, the presentation pattern might just reflect access to healthcare. In many European countries, there are affordable eyecare provisions, such as the NHS [45], whereas in the US, there is a scarcity of such eye services [46]. This may create barriers to eye care, such as cost and insurance [46]. Most studies included in this review were conducted in the US. Several individuals in these studies displayed advanced disease, possibly due to a lack of early diagnostic option due to barriers to attending eye services.

### 4.1. Ocular Complications

The most commonly reported retinal findings in C3DDD patients were drusen deposits and CNV. This indicates that, despite many patients remaining asymptomatic, several patients experience severe ocular complications. Retinal drusen can be considered a normal part of aging, and many older people will have some present. Large-sized drusen and large quantities could indicate age-related macular degeneration. Sometimes, a complication of these drusen deposits can be neovascular membranes, which may form from oxidative stress and inflammation resulting from BM damage [4]. Other eye diseases included CSCR and hypertensive retinopathy. These eye diseases were often deemed unrelated to C3DDD. CSCR predominantly affects men with Type A personality, and this was reflected in the current review where 75% of CSCR affected males [47]. There could be a link to CSCR and renal transplant, which is often accompanied by the use of steroids to reduce risk of rejection; however, only one paper with CSCR reported kidney transplantation, and therefore we do not know. The prevalence of eye diseases across included studies was inconsistent. One study noted that all participants had nyctalopia (night blindness) [39]. Despite a 100% occurrence rate in this study, nyctalopia was rarely documented in other included studies, perhaps due to clinicians not asking about the symptom.

Drusen is the most well-documented retinal pathology associated with C3DDD [3]. In this review, drusen was the most common retinal pathology by a considerable margin. Deposits were primarily in subretinal and macular regions. The definite cause of deposits was not identified. The RPE and glomerular podocytes both generate complement factor H (CFH) [4]. Therefore, CFH mutations causing GBM deposits could also cause retinal deposits [4]. Other ocular complications were commonly highlighted, including RPE detachment, atrophy, and haemorrhaging. Changes in drusen appearance were commonly associated with disease duration. Many individuals with a longer disease duration had more numerous drusen [28], and this was supported by other studies stating drusen size increased with time [4]. Often, the macular area was consistent with the area containing the highest drusen density [38]. This may reflect why the current review found both a high density of macular drusen and a high occurrence of macular atrophy. However, not all studies reported a connection between drusen and disease duration/severity. One individual had CSCR and RPE detachment [15]. Despite presenting advanced ocular symptoms, they displayed no drusen throughout the disease course. Additionally, in a study of eight individuals, only one was found to have ocular drusen, despite all individuals having an average evolution time at examination of 19 years [10].

Multiple studies questioned whether renal transplantation exacerbates ocular complications. Within a study of 26 patients, the only patient presenting CNV was also the only transplant recipient [34]. Another patient developed severe ocular complications 6 months post renal transplant, including CSCR and haemorrhaging [19]. In 1992, ocular impacts of organ transplantation were first analysed [48]. Five transplant recipients developed visual loss and demonstrated RPE clumping. Despite these findings, it remains unclear whether transplantation causes visual defects in C3DDD, highlighting areas for future research.

### 4.2. Management and Treatments

There is no current cure for C3DDD, and it is primarily managed systemically with blood pressure control and reducing protein in the urine, with some individuals also receiving kidney transplants [1,5].

The literature states that there is a weak relationship between the severity of C3DD and retinal complications; therefore, systematic treatments [49,50], steroids, and liver transplants [50] tend to have little effect on the retinal drusen caused by C3DDD [50,51]. It is unclear if the C3DDD directly affects or impacts on the progression of other retinal complications such as CNV.

Many of the papers do not report on treatments given to patients within the case reports; this is likely due to no treatment being available for the most common complication, retinal drusen deposits. Some studies report treatment with injections and laser but only where there is a CNV or retinal fluid. There was likely a switch from laser to anti-VEGF around 2004/2005 when anti-VEGF was first introduced. This would be considered the first line treatment for neovascularisation at present. Patients who lose vision can also avail of low vision support.

These treatment findings from the papers is are in line with the literature which states that there are local treatments to help with some of the retinal complications (whether they are related to C3DDD) such as neovascularisation [32], usually with an improvement in VA [52]. Emerging treatment for dry AMD could be effective in treating the retinal drusenoid deposits described in the future.

Management of ocular and retinal complications in C3DDD likely varies between countries according to clinical capacity, multidisciplinary teams, and other factors. It is suggested that people with a diagnosis of C3DDD should be assessed by an ophthalmologist at the time of diagnosis and monitored regularly through retinal imaging including fundus imaging and slit lamp examination [39].

Despite this, some countries do not have the clinical capacity to do this, and many do not get visual symptoms or vision-altering complications. It is therefore important for nephrologists to be aware of the risk of retinal and visual changes in order to explain this to their patients as they are not regularly seen in ophthalmology clinics. Nephrologists should advise patients to report any visual symptoms, in particular distortion, immediately to their health team and refer to ophthalmology for assessment.

### 4.3. The Role of Complement Factor H (CFH)

The complement system, a group of proteins, works to fight infection. The system can sometimes become dysregulated which can lead to complement components being deposited [53]. Complement Factor H is specifically designed to be a regulator of the alternative pathway of complement activation. In C3 DDD, genetic mutations or other factors impairs its ability to regulate this alternative pathway, causing extensive deposits on the kidney that lead to inflammation and kidney damage [53,54].

While this is happening in the kidneys, CFH can also affect the eye and is specifically associated with age-related macular degeneration (AMD) [55]. It is thought that around 50% of AMD cases had CFH polymorphisms present [55]. Age-related macular degeneration has a known association with vascular pathology [56,57,58,59,60], and the pathogenesis of this is associated with Bruch’s membrane thickening and reduction in flexibility [61,62,63], which ultimately leads to age-related lipid deposits accumulating within the retina, restricting its blood supply, resulting in Geographic Atrophy (GA) which can progress to CNV [55]. This is likely the reason that these drusenoid deposits appear in the retina for C3DDD patients and resemble AMD. These drusenoid deposits are much like those in AMD and can cause visual problems in more progressed stages; these cannot be treated at present. These deposits can also progress to CNV, much like in AMD, which can be treated with anti-VEGF.

### 4.4. Strengths

This scoping review followed a robust and transparent methodology, aided by a framework such as PRISMA-ScR. The selection process used a dynamic team approach. This review included diverse study designs and employed no limitations regarding patient population. This resulted in a unique large dataset, providing an unprecedented opportunity to comprehensively map data. Furthermore, charting data enabled the identification of gaps in the current literature. This review highlighted that the exact clinical manifestations of C3DDD are unclear and therefore provides a foundation for future research.

### 4.5. Limitations

Most papers included were case reports and 67% described only one patient, reflecting a lack of large sample sizes and generalisability. Case reports typically focus on unusual or rare cases rather than common disease occurrences. Therefore, it is possible that this review overrepresents symptoms that are uncommon in the general population. There are some queries around terminology differing between countries and cohorts around retinal findings RPE elevation, RPE detachment, and Bruch’s membrane irregularities; however, without clear definitions, it is unclear how these are similar and how they differ, and we have therefore reported them as they are written within each paper.

## 5. Conclusions

In conclusion, patients with C3DDD are at risk of retinal complications, primarily drusen-like deposits. There needs to be further research into the etiology of ocular complications and the effect of long-term disease and transplantation on ocular complications and symptoms.

## Figures and Tables

**Table 1:** Geographical distribution of studies.

Country of Study	n (%)
United States	21 (55%)
Europe	17 (45%)
**Geographic Breakdown of European Studies**	
England	9 (24%)
Germany	3 (8%)
Netherlands	2 (5%)
Spain	1 (3%)
Switzerland	1 (3%)
Turkey	1 (3%)

**Table 2:** Visual Acuity data (VAs across case studies).

LogMAR	Total	%
0.0	43	40.19
0.1	7	6.54
0.2	15	14.02
0.3	19	17.76
0.4	2	1.87
0.5	2	1.87
0.6	1	0.93
0.7	1	0.93
0.8	1	0.93
0.9	1	0.93
1.0	4	3.74
1.2	1	0.93
1.5	1	0.93
Counting Fingers (CF)	3	2.80
Hand movements (HM)	1	0.93

**Table 3:** Eye disorders.

Eye Disorders	n (%)
Hypertensive Retinopathy	7 (7%)
Central Serous Chorioretinopathy (CSCR)	6 (4.7%)
Retinoschisis	2 (1%)
Glaucoma	1 (0.7%)
Amblyopia	2 (1%)
Macular Degeneration (undefined)	2 (1%)
Purtscher-like retinopathy	1 (0.7%)

**Table 4:** Clinical features identified.

Clinical Features	n (%)
Drusen-like Deposits	103 (75%)
RPE Detachment	24 (18%)
Choroidal Neovascularisation	22 (16%)
Macular Atrophy	17 (12%)
Retinal Haemorrhage	15 (11%)
Bruch’s Membrane Irregularities	15 (11%)
RPE Elevations	13 (9%)
Subretinal Fluid	10 (7%)
Intraretinal Fluid	2 (1%)
Retinal Atrophy	7 (5%)
RPE Mottling	5 (4%)
Retinal Pigment Migration	4 (3%)
**Retinal Vascular Changes**	3 (2%)
**Maculopathy**	2 (1%)
**Cotton Wool Spots**	2 (1%)
**Macular Oedema**	1 (0.7%)

## Data Availability

The original contributions presented in this study are included in the article. Further inquiries can be directed to the corresponding author.

## Appendix A. PRISMA SCr Checklist

**Table:**

**Section**	**Item**	**PRISMA-ScR Checklist Item**	**Reported on Page #**
**TITLE**			
Title	1	Identify the report as a scoping review.	1
**ABSTRACT**			
Structured summary	2	Provide a structured summary that includes (as applicable): background, objectives, eligibility criteria, sources of evidence, charting methods, results, and conclusions that relate to the review questions and objectives.	1
**INTRODUCTION**			
Rationale	3	Describe the rationale for the review in the context of what is already known. Explain why the review questions/objectives lend themselves to a scoping review approach.	2
Objectives	4	Provide an explicit statement of the questions and objectives being addressed with reference to their key elements (e.g., population or participants, concepts, and context) or other relevant key elements used to conceptualize the review questions and/or objectives.	3
**METHODS**			
Protocol and registration	5	Indicate whether a review protocol exists; state if and where it can be accessed (e.g., a Web address); and if available, provide registration information, including the registration number.	3
Eligibility criteria	6	Specify characteristics of the sources of evidence used as eligibility criteria (e.g., years considered, language, and publication status), and provide a rationale.	3–4
Information sources	7	Describe all information sources in the search (e.g., databases with dates of coverage and contact with authors to identify additional sources), as well as the date the most recent search was executed.	3
Search	8	Present the full electronic search strategy for at least 1 database, including any limits used, such that it could be repeated.	3
Selection of sources of evidence	9	State the process for selecting sources of evidence (i.e., screening and eligibility) included in the scoping review.	3–4
Data charting process	10	Describe the methods of charting data from the included sources of evidence (e.g., calibrated forms or forms that have been tested by the team before their use, and whether data charting was done independently or in duplicate) and any processes for obtaining and confirming data from investigators.	4
Data items	11	List and define all variables for which data were sought and any assumptions and simplifications made.	4
Critical appraisal of individual sources of evidence	12	If done, provide a rationale for conducting a critical appraisal of included sources of evidence; describe the methods used and how this information was used in any data synthesis (if appropriate).	n/a
Synthesis of results	13	Describe the methods of handling and summarizing the data that were charted.	4
**RESULTS**			
Selection of sources of evidence	14	Give numbers of sources of evidence screened, assessed for eligibility, and included in the review, with reasons for exclusions at each stage, ideally using a flow diagram.	4
Characteristics of sources of evidence	15	For each source of evidence, present characteristics for which data were charted and provide the citations.	4–9
Critical appraisal within sources of evidence	16	If done, present data on critical appraisal of included sources of evidence (see item 12).	n/a
Results of individual sources of evidence	17	For each included source of evidence, present the relevant data that were charted that relate to the review questions and objectives.	4–9
Synthesis of results	18	Summarize and/or present the charting results as they relate to the review questions and objectives.	4–9
**DISCUSSION**			
Summary of evidence	19	Summarize the main results (including an overview of concepts, themes, and types of evidence available), link to the review questions and objectives, and consider the relevance to key groups.	9–10
Limitations	20	Discuss the limitations of the scoping review process.	11
Conclusions	21	Provide a general interpretation of the results with respect to the review questions and objectives, as well as potential implications and/or next steps.	11
**FUNDING**			
Funding	22	Describe sources of funding for the included sources of evidence, as well as sources of funding for the scoping review. Describe the role of the funders of the scoping review.	15

n/a = not applicable, # = number.
